# Collectanea

**Published:** 1833-03

**Authors:** 


### COLLECTANEA.

**Floriferis ut apes in saltibus omnia libant,**
**Omnia nos, itidem, depascimur aurea dicta.**

### PATHOLOGY.

*Case of Tetanus, following a Punctured Wound in the Foot, cured by Division of the Posterior Tibial Nerve.* By JOHN MURRAY, M.D. Assistant Surgeon, Hon. E. I. C. Service.

I WAS a passenger in the ship *James Pattison*, from England to Calcutta, when, in the channel, three days after leaving Portsmouth, at ten o'clock A.M. on the 15th August last, the surgeon of the vessel (Mr. D. Leslie) came and reported to me that Mr. William Pile, one of the midshipmen, æt. fifteen, had trod upon a rusty nail the preceding evening about nine o'clock, which penetrated the left foot, between the metatarsal bones of the great toe and the adjoining one, and that symptoms of locked jaw had made their appearance. The patient had kept his watch after the accident during the night, which was cold, and the wound had been very painful. When he was seen in the morning at eight A.M. he complained of a stiffness about his jaws and throat, which had increased very much since that time. His countenance was anxious, and his lips appeared swollen and rather livid. A poultice to the wound was the only treatment that had been employed. On consultation, the following draught was agreed to be given:—℞ Pulv. Camphor. ʒss. Tinct. Opii, ♏ lxx.; Syrup. simpl. ℥j. M. s.s.

And as the jaws were nearly closed, and great difficulty found to get them opened to the extent of a quarter of an inch, a piece of wood of this thickness was inserted between the teeth. Half an hour after taking the draught he was visited again, but no beneficial effect had resulted from the opiate. The tetanic symptoms were rather increased, the spasms had partially extended to the muscles of the neck, and the piece of wood was deeply indented by the teeth. The limb was cold, and he said "it was dead, excepting at the site of the wound, which was painful, and that he had little power of moving it." His pulse was 120, and what may be called irritable; and his situation seemed to be one of great danger. He was ordered to be carried to one of the best cabins; and after considering all the different modes of treatment that have usually been recommended and employed in tetanus, and finding how undecidedly they were all spoken of as to affording hopes of cure, I proposed to Mr. Leslie the division of the posterior tibial nerve (by which the injured part was supplied) as a remedy that held out a good prospect of success, from its cutting off the communication between the source of irritation and the brain, at the same time that it was an operation easily performed, and unattended with danger or deformity. I proposed, also, that the original wound should be dilated and cauterized.

My proposals being agreed to, as soon as the necessary preparations could be made, the operation was performed. A straight incision, an inch and a half in length, was made through the integuments and the aponeurotic fascia, about an inch posteriorly to the malleolus internus, which laid bare the sheath of the vessels; and on dissecting deeper, I easily found the nerve in its usual position. By an aneurism needle I separated and raised the nerve, so that I might divide it with greater facility and expedition. When brought thus into view, it appeared to be so remarkable large, (being nearly twice the usual size) though of the natural colour, that a doubt was expressed by Mr. Leslie about its really being the nerve. To satisfy him on this point, I requested the patient to extend his foot, which he did with difficulty, and in an imperfect manner, as he said he had nearly lost the power of moving it; but he did it sufficiently to shew the difference between the nerve and a tendon, as it did not become tense in this action. The nerve was then rapidly divided by a single cut of the scalpel, which gave acute pain; and although he could not articulate distinctly before, on account of the closed state of his jaws, *he immediately opened his mouth with an exclamation;* and, on looking at his countenance, I was astonished at the striking improvement in it. I asked him how he felt, and he said "he was already much better, and that his leg had come to life again." He expressed at the same time a great inclination to go to stool. There was scarcely any hemorrhage from the wound, which was then simply dressed by bringing the edges together by adhesive plaster, with lint, and a bandage over it. We next dilated the original wound made by the rusty nail, which also gave (rather unexpectedly) great pain, but did not cauterize it as proposed, as the symptoms had already yielded. A poultice, sprinkled with laudanum, was applied over it. His bowels were then copiously moved; and on being placed in bed, without any additional opiate, he fell into a sound sleep, which lasted without interruption for four hours, and he awoke very much relieved from all his former disagreeable symptoms. His jaws still, however, felt rather stiff, but by a strong exertion he could open them nearly to the full extent. The excessive pain in the original wound, of which he complained previously to the operation, had now entirely ceased, and the motion of the limb was quite restored. It was found, on examination, that the heel and sole of the foot were quite benumbed, but the sense of feeling in the upper part of the foot was not affected. At night he had the following powder:—℞. Pulv. Opii, gr. ij.; Pulv. Camphoræ, ℈j. M.

Aug. 16th. Slept indifferently during the night; complained still of the stiffness of the jaws and neck, and to a greater degree than the preceding evening. He also had pain and tightness across the chest, with headach. I was very sea-sick, and in bed by this time, and unable to make more minute inquiries about the symptoms, but I recommended venesection and another opiate to be given. He was bled accordingly to the extent of twelve ounces; which induced syncope, and gave great relief; and he had the same opiate as last night, after which he slept a good deal during the day. His bowels were naturally opened, and the same opiate was repeated at bedtime.

17th. The opiate of last night appeared to produce considerable excitement for some time after it was administered, but this was followed by sound sleep. The stiffness of the jaws and neck, and tightness across the chest, have nearly altogether disappeared. He complains of a great deal of numbness in the leg and foot. Pulse eighty, and natural; tongue clean; bowels regular.—Rep. Pulv. h. s.

18th. The tetanic symptoms have entirely disappeared; the numbness of the leg is gone off, and he complains of pain in it, extending from the knee downwards. The parts in the site of the operation are very tender, and extremely painful on pressure. The original wound continues to be poulticed, and looks healthy. The opiate powder to be continued in half the former quantity.

From this date no unfavorable symptoms occurred. The wound in the sole of the foot healed in a few days; that of the operation did not unite by the first intention, and did not heal till about a fortnight after. The sense of feeling began to return in the sole of the foot on the third day after the operation, and is now natural in most places; but there is still, up to this date, an entire want of sensation in the little toe and heel. He complained for some time of not being able to perform the action of separating the toes so easily as formerly, but that no longer exists. He finds no inconvenience in walking, or in the performance of all his regular duties: in fact, he is quite well.

*Remarks.* I have thought the above case worthy of notice, from the result of the usual modes of treatment pursued in this disease (as given by our best authors) having been so generally unsuccessful. Hennen, whose experience in it must have been great during the Peninsular war, says, "Happy should I be if I could afford any thing satisfactory on this complaint; but in truth, my observations have tended more to show me what I could not trust to than what I could place the smallest reliance on, when the disease was once fully formed." And he further adds—"I have never been fortunate enough to cure a case of acute symptomatic tetanus." I think it important to notice that he mentions, that in one case he found, on dissection, the nerve connected with the injury thickened. Seeing this appearance so remarkable in the instance I have recited, the lengthened account of tetanus in Cooper's Surgical Dictionary, which I consulted at the time, rather bewildered me as to the treatment to be followed, and only showed the great difficulty to ascertain, amongst numerous discordant accounts, what practice seemed to be attended with the least ill success. "Nothing," as he says, "is a more certain proof of our not being acquainted with any effectual method of treating a disease than the multiplicity of remedies recommended, which are as opposite as possible in their effects." I decided upon following the indication which seemed to me the most rational, and not to adopt any plan or to use any remedies proposed on empirical principles, seeing how unavailing they had mostly all been. I am only aware of one instance in which the communication was cut off between the wounded part and the sensorium, by a suitable division of the nerve supplying the part. It is one adduced by Baron Larrey; and, as in that case the operation was followed by a similar result to the present, I am surprised that the hint never was taken from it, and followed up by others. Amputation has frequently been performed with the same view, but has seldom succeeded, probably from its having been too long delayed; and I would ask why such a severe and maiming operation should ever be thought of, or persisted in, when the same object can be so much more readily and scientifically attained by a simple incision?

We are told that, when the disease invades very quickly, the danger is always greater than when it comes on at a more distant period from the receipt of the injury. In this case its attack was very early, and the progress of the symptoms rapid; and I have no doubt that it would soon have run its course to a fatal termination, if it had been left to the sanative powers of nature alone, or treated in the usual manner; but very little time was lost in having recourse to the operation that proved a remedy of immediate and effectual power, after the disease had decidedly formed; and I think it may have been of the greatest consequence that it was resorted to so soon after trismus appeared.—*Med. Gazette.*

---

### PRACTICAL MEDICINE.

*Observations on the Use of the Hydrocyanic or Prussic Acid in Pertussis, or Hooping Cough.* By Edwin P. Atlee, M.D. of Philadelphia.

From the numerous cases in which I have prescribed the acid in hooping cough, and an attentive observation of its powers in this appalling disease, when unattended by any other, I confidently recommend it to the medical profession. In no instance has it proved deleterious to my patients, but rather seems to have destroyed a previous predisposition to croup and catarrh.

The following is the course pursued. During the first, or what may be termed the inflammatory stage of the disease, I resort to the general depletory agents, if called on at all. Usually, however, parents seldom apply for medical advice until the second, or spasmodic stage, or that in which the hoop is clearly discerned. If, on inquiry, the bowels have not been freely evacuated, a full dose of calomel and rhubarb, according to the age and condition of the patient, is prescribed. If, to use the common phrase, "the child is much stopped up with phlegm," emesis is produced by antimonial wine, which, when judiciously prepared, I prefer to any other emetic. After this, the syrup is given as follows:

For a child six months old, one drop of the acid to one ounce of simple syrup; a tea-spoonful twice a day. If no uneasiness, dizziness, or sickness, is produced within forty-eight hours, the same quantity is given three times a-day. From six months to a year, the same may be given four times a day.

| | | | | | |
|---|---|---|---|---|---|
| From 1 | to 2 | years of age, Hydrocyanic acid, | gtt. ij., | Syrup, ℥i. | |
| 2 | 3 | do. | do. | iij. | do. |
| 3 | 6 | do. | do. | iv. | do. |
| 6 | 12 | do. | do. | v. | do. |
| 12 | 15 | do. | do. | vj. | do. |
| 15 | 20 | do. | do. | vij. | do. |
| 20 | 30 | do. | do. | viij. to x. | do. |

A small or large tea-spoonful being the dose in each case, repeated as often as close observation of its effects will warrant. I have never yet given it more than four times a-day.

Having tried the imported acid, and found it entitled to little reliance, I have for the last six years prescribed that prepared in this city by our judicious chemists, Furr and Kunzi, according to the formula of Mr. Brande. It contains four and a half per cent. of pure prussic acid of Gay Lussac, and is therefore not so strong as that recommended by M. Majendie.

Believing that the acid might be efficacious in spasmodic asthma, I requested my young friend, Dr. Isaac Parrish, late resident student of the Philadelphia Alms-house, to try it. He accordingly prepared the syrup in the proportion of four drops to the ounce, and gave a tea-spoonful every two hours, with decided benefit, The patient experiencing great relief in a few hours. This case, he states, to have resisted any impression from the other ordinary remedies.—*American Journal of Medical Sciences.*

---

*Cure of Tic Douloureux by Stramonium.* A young lady, who had suffered for many years from tic douloureux, (the attacks of which terminated sometimes by the swelling of the cheek or of the lip of the affected side,) had employed a number of remedies, without success. Dr. Pott cured her in the space of six weeks, by applying the actual cautery to the arm, and giving her, internally, eight or fifteen drops of tincture of stramonium every three hours.—*Beitraege Mecklenburgischen Aertze*, and *Dublin Journ. of Med. and Chem. Sciences.*

---

*Sulphur as a Preservative against Measles.* Dr. Tourtual, a Dutch physician, states that, at a period when measles were epidemic, all the children who were under treatment with sulphur for itch escaped the disease; and that those who were taking sulphur for the cure of hooping cough enjoyed the same immunity. Finally, he says that many children who were given a mixture of sulphur and camphor, and to whom these medicaments were applied by frictions, were not attacked with measles, whilst those who were not subjected to that medication were affected. —*Kleinert's Repertorium*, and *Gaz. Médicale*.

---

*Leucorrhœa*. Dr. Kopp, in a late number of *Hecker's Annalen*, recommends the following mode of treatment of leucorrhœa, which he says he has frequently employed with advantage. A piece of sponge, of proper size to fill completely the vagina, is to be dipped into the following solution and introduced into that canal at night before going to bed. ℞. Decoc. Ratanhiæ, ℥xij.; Ext. Ratanhiæ, ℥ss.; Tinct. Catechu. ʒiss.; Tinct. Kino, ʒiss. M.

Dr. Cless, in the *Archiv. fur Medizin Erfahrung*, states that he cures almost all the cases of leucorrhœa that occur in this hospital at Stutgard with cubebs.—*Gaz. Med.*

---

*Hysteria*. Professor Chiappa states, that enemata of iced water immediately dissipate the symptoms which characterize the hysteric paroxysm.—*Annali Universali.*

---

### PRACTICAL SURGERY.

*Utility of Malt Poultices*. In a recent number of the *American Journal of Medical Science*, Dr. Williams relates several very interesting cases of severe and offensive ulcerations, in which the malt-poultice was applied with great and rapid advantage. He prepares it in the following manner: Stir into good boiling beer as much ground and sifted malt as will be sufficient to form it into a poultice of a proper consistence; spread it thick upon cotton or linen cloth, and cover the surface of the poultice with about a table-spoonful of the best yeast, and lay the poultice thus prepared upon the ulcer. Spread the poultice much larger than the ulcer, and change it two or three times in the course of twenty-four hours. When barley-malt cannot be obtained, oat-meal will answer. The ulcer will be sweetened, and will discharge bland matter in twenty-four hours; yet it will be advisable to continue the poultice three or four days.

We think, with Dr. Williams, that many substitutes for the malt-poultice have lately been introduced into surgical practice, which are much less efficacious in ill-conditioned and sloughing ulcers, and even in ulcers which have assumed a gangrenous appearance.

---

*On the Application of the Actual Cautery in Vesico-Vaginal Fistula.* Condensed from a paper by Dr. Kennedy, in the *Dublin Journ. of Med. and Chem. Sciences*, Nov. 1832.

A fistulous communication between the bladder and vagina inflicts upon the patient the greatest distress and vexation, and not unfrequently the surgeon finds great difficulty in effectually relieving the disease. This injury is often produced by mismanagement during labour; the accoucheur either too long delaying the delivery of his patient, until the pressure produces destruction or sloughing of the parts, using instruments violently, or forcing the catheter through the urethra or neck of the bladder; and, secondly, in these affections being considered by many, if not irremediable, at least to promise so little chance of cure, as to induce them to try only palliative treatment. The worst case Dr. K. ever met with was produced by violence *in primo coitu*, in a newly-married woman: the vagina and bladder formed one cavity; and so completely was the septum destroyed as to preclude the possibility of relief by any operation.

Various plans have, at different times, been recommended with a view to the cure of this distressing complaint. By some, the introduction of a catheter into the bladder, and the detention of it there for some time, has been supposed sufficient for this purpose. Others have recommended that pressure should be made over the aperture, with a view to prevent the urine escaping through it, and to cause it to close; and a host of sufficiently ingenious contrivances have been invented to endeavour to produce this effect: of these, the elastic bottle, made to fit within the vagina, appears to answer best. Again, a combination of these plans of treatment has been recommended; and this constituted Desault's method, which was long adopted in the schools. Experience, however, has unfortunately proved that these plans, although serviceable in slight cases, and where had recourse to immediately after the occurrence of the accident, are quite unavailing in cases of long standing, or where the opening is considerable. More enterprising practitioners have attempted to produce a union of the edges of the aperture; and with this view, after paring them with a knife or scissors, have fastened them together by means of sutures. The difficulties in this operation are very considerable; whilst, to afford a likelihood of success, it is frequently necessary to repeat it again and again. Mr. Earle speaks of his performing the operation so often as thirty times in the same case, before success crowned his efforts. Now, although an occasional cure is undoubtedly effected by the suture, yet the difficulties* and fatality attending it oppose such obstacles to the operation, that medical men are, in the great majority of cases, deterred from having recourse to it, deeming it preferable to resign the unhappy female to a life of wretchedness, and leave her an object of disgust, not merely to herself, but to all those about her,

* Mr. Earle, in his lecture on the operation of suture in vesico-vaginal fistula, points out these difficulties in very strong and correct language: we shall here merely enumerate them. The narrow space for operation, continual flow of urine, which blunts the delicate instruments necessary; great sensibility of the parts engaged, their disposition to slip from the instrument; the impression made on the pelvic viscera by a cough or sneeze; and great indisposition in the mucous surfaces to take on adhesive inflammation.—See *Medical Gazette* for November 14, 1829.

than to risk having recourse to a hazardous operation, with (at best) but a very questionable* chance of success. Caustics and stimulating ointments have been tried for the purpose of promoting union of the fistulous edges: the results attending their application have been quite as unsatisfactory; while the difficulty of confining their operation to the margins of the aperture, and the risk of their injuring the interior of the bladder, in a great measure preclude their use.

The actual cautery, as practised and recommended by Dupuytren,† appears to hold out a fair promise of relief, without risk, and even a likelihood of effectual cure in these distressing cases. At first, it might appear that the difficulties attending the application of a red-hot iron within the vagina would be very considerable, and that it could not be accomplished without much suffering to the patient, and risk of injury to the internal parts; in forming this opinion, we would be in error, as it may with very little management be effected, and that with perfect safety. There being some omissions, and a want of attention to certain precautions, in the account which we find in the Journal Hebdomidaire, of this operation, as practised by Dupuytren, I shall describe the manner in which I have usually performed it.

The instruments requisite are, a flat female catheter, two female sounds, a speculum, and cauterising iron. Where the aperture is very high up in the bladder, the speculum I prefer is the large two-bladed pewter one used by the French; as Weiss's small three-bladed speculum, which answers in ordinary cases, when the lesion is more within our reach, does not, in the former, so well expose the part, or protect the vagina from the iron. Where the opening is in the neck of the bladder, or urethra, the operation may be quite as well performed by dilating and protecting the vagina with curved spatulæ. In an operation of this kind, in which I lately assisted Dr. M'Dowel, three broad brass spatulæ, one introduced towards the perineum, and one at each side of the vagina, exposed the lesion remarkably well: this plan has the advantage of not pushing up the bladder further into the vagina, an inconvenience which it is next to impossible to avoid with the speculum. The cautery should be as nearly as possible the shape of the opening in the bladder, but somewhat larger. That which we shall generally find to answer is one of an oval shape, with its longer diameter placed transversely. As in the majority of cases, the longest measurement of the aperture is from side to side, and in such the difficulty of affecting an union is greatest; in some cases, however, the aperture has its longest measurement from before backwards, and then our cautery must be similarly constructed. The margin of the cautery ought to be rather more raised than the centre, as our object is to touch the

* Mr. Earle succeeded in curing only three cases in this way, although he speaks of so many as twenty-one patients afflicted with this disease having come under his notice.— *Med. Gaz.*

† Journal Hebdom., No. 58.

edges of the fistula without injuring the mucous membrane of the bladder.

In applying the cautery, we should place the patient lying forwards upon a table, with her limbs hanging over the ends, which should be near a window; elevating the pelvis upon bolsters or blankets placed under it. The limbs should then be separated, and the light thrown as much as possible into the vagina. Where sufficient light cannot in this way be procured, a candle must be used. The speculum is to be introduced, and the lesion brought into view; a flat female catheter must now be passed through the urethra, and placed across the opening, within the bladder, taking care, at the same time, to reduce any protrusion of the vesical mucous membrane, and retain it out of the reach of the cautery. When the opening into the bladder is very considerable, or the catheter is insufficient, it may be necessary to pass a second instrument through the urethra to effect this object. I have found the introduction of two female sounds answer remarkably well, where a second instrument was necessary. As folds of the vaginal mucous membrane sometimes protrude between the blades of the speculum, the operator must guard against this, and examine whether the instrument be so adjusted as to prevent the vaginal passage being injured by the iron; taking care that the interior of the bladder is well protected, and the edges of the aperture completely within his reach. Having satisfied himself in these respects, he is carefully to introduce the cautery, heated to a white heat, and, having steadily touched the edges of the fistula, to withdraw it and introduce a pledget of lint dipped in cold water, after which he may gradually remove the speculum. The cautery must only touch the part, for if retained too long in contact with it, might produce a sloughing eschar.

The operation is extremely simple, and performed in a minute; and although the name of the actual cautery sounds rather harshly, its application is in my mind, by no means as severe, or attended with such suffering, as the suture. The patient should be put immediately to bed, and strict quiet enjoined. The after-treatment necessary is merely to keep the bowels gently open, and pay attention to the abdomen, lest tenderness should set in: this I have but once known to occur after the cautery; and then it was relieved, immediately, by warm stuping and aperients. After the immediate irritation has subsided, it is necessary to introduce a gum elastic catheter into the urethra, and retain it in the bladder, to ensure the urine's passing through it; a precaution the more called for, as, in some cases of long standing, this passage has become so collapsed as to be almost impervious. However, in cases where the catheter, on being introduced, passes through the fistula into the vagina, it is better not to leave it in the bladder, else it will do more harm than good. In such it will be sufficient to pass it into the bladder once or twice a day, to endeavour to restore the urethra and bladder to their proper state, by bringing the urine in its natural direction. It is necessary that the patient should retain the recumbent posture, and remain perfectly quiet while under treatment; and this point cannot be too much insisted upon. We should also ascertain in what position, namely, whether lying on the back, on one or other side, or the abdomen, she can retain her urine for the greatest length of time, and cause her to remain in that position as much as possible. This has two recommendations: in the first place, it prevents the urine from constantly escaping out of the fistulous aperture, which interferes with the union of its margins, and progress towards a cure; and in the second, by retaining the urine as long as possible, the bladder, which from being continually empty, had become contracted, is, by this means, gradually distended, and rendered more capable of fulfilling its office of reservoir. By attending in this way to position, and the situation of the fistula, the bladder will be induced to contain a considerable quantity of water, in which it is assisted by the transverse distention observed in it as the effect of pregnancy. It is very desirable, however, with a view to recovery, that the urine should be induced to pass through the urethra, and not by the fistula; and, if the plan here recommended interfere with this, it is better to let the patient assume the position in which the urine escapes through the urethra, if it does so more in one than another.

The effect of the cautery is to produce a thickening of the margins, and consequent contraction and diminution of the aperture, and ultimately an adhesion of its edges, closing it up altogether. Upon the size and position of the aperture will depend the greater or less likelihood of perfect cure.

The operation may require to be several times repeated. Whether by persisting in repeating it sufficiently often, we should, even in the majority of cases, succeed in closing the aperture, I cannot say, but rather think not. Fortunately, however, it does not require that the aperture should be actually closed to enable our patients to retain their urine, as a very good substitute for the adhesion of the sides of the fistula occurs in the extension of its margin or lip across the aperture, thus forming a kind of valvular closure of it, by which means the bladder becomes capable of retaining the urine almost as well as if the opening were closed. In a patient whom Dr. Green saw with me, this effect was produced in a striking degree; and although her urine was constantly escaping from her before the cautery was had recourse to, she was enabled afterwards to retain it without difficulty for six or seven hours. In a case which Dr. Collins saw with me, although the operation was performed six times, yet the aperture did not completely close; but the thickening of the margin of the fistula took place, in consequence of which the woman was able to retain her urine throughout the entire night, and for several hours (even when walking and using active exertion) during the day, although on her coming to me it was constantly escaping.

With these proofs, then, of the utility of this plan of treatment, I can have no hesitation in recommending its adoption to those whose opportunities afford them the power of putting it to the test, trusting that further experience may add, to greater facility in its performance and perfect freedom from danger, the two advantages which it undoubtedly possesses over the needle, that most important advantage of all, a greater certainty of cure.

Dr. M'Dowel states, in a letter to Dr. Kennedy, that he has "tried the actual cautery in two instances of vesico-vaginal fistula with considerable success."

---

*Hypertrophy of the Tongue*, by Dr. Wells. (*American Journal of Med. Sciences.*)

A daughter of George Roberts, of Lexington, aged six years, was brought to Columbia for professional assistance in May, 1829, with an enormous enlargement of the tongue; otherwise she was in good health, and a fine robust girl.

The following are the dimensions and state of the tongue at the time: length as it remained at rest and hung down over the chin, from the superior incisors to its apex, two and a half inches; circumference just in front of the lips, six inches; breadth from one angle of the mouth to the other, a little more than two inches. It had undergone a very considerable change in structure, was much more dense than natural, and not subject to change in its dimensions by the action of its own muscles, or, if at all, very slightly so; its motions otherwise were sufficiently free; upper surface smooth; inferior covered with the cicatrices of old ulcers, several of which, where the tongue rested upon the alveolar processes of the lower jaw, but imperfectly healed; colour darker than natural. Within the mouth the tongue had undergone no apparent change, except a moderate increase in width and thickness. She had formerly suffered much from inflammation and ulceration of the mucous membrane of the tongue, but this difficulty had been obviated for the last six or eight months by keeping the organ covered with cloth bags, which, becoming immediately saturated with the mucous secretions of the parts, afforded a complete protection from the external air. If these bags were omitted for a few days, the surface became very sore and painful. The front teeth had been displaced from the lower jaw by the long-continued pressure of the tongue. The lower lip was folded downwards. The anterior portion of the superior maxillary bone had undergone a slight curve upwards, the inferior a much greater curve downwards, so that, while the back teeth came in contact, the front were an inch asunder, or rather the space between the upper teeth and the corresponding alveolar processes below was something more than an inch. She managed to eat by placing the morsel of food between the back teeth with her finger; fluids were introduced into the mouth through a tube, such as the spout of a coffee-pot: could articulate with a good deal of distinctness.

We could obtain nothing very satisfactory of the history of this case from the father, by whom she was accompanied. Her mother had died during her early infancy, and she had been placed with her grandmother away from him, the parties not of the most intelligent class. It seemed, however, from the father's statement, that the difficulty commenced when she was about eighteen months of age, with an attack of what we suppose to have been common glossitis: the tongue became suddenly swollen, and protruded from the mouth, continued in that state for two or three weeks, when the swelling gradually subsided, and it was again brought within its proper limits. During the next two and a half years she had repeated attacks of a similar character; worse, and of a longer duration, in cold weather. All that he could say of the constitutional symptoms at this period, was, that "when the tongue swelled the child cried a great deal, and was very sick; and the old lady was in the habit of dosing her frequently with salts."

For the last year or two there seems to have been no particular change in the dimensions of the tongue, save a gradual increase corresponding with the general growth of the system.

Not considering it within the scope of medical science to bring the tongue back to its normal state, and being confirmed in this opinion by that of several of my medical friends, the removal of that part external to the mouth was proposed and acceded to, and would have been done by one stroke of the knife, but for the refractory character of the patient and the timidity of the father. The following plan was resorted to, and is on the whole perhaps the best operation in such cases and with such patients; but, in adults, where the surgeon can have the convenient use of all his resources for controlling hemorrhage, there can be no objection to free excision, the least painful mode of operating.

The child having been freely purged, and kept on a gruel diet for two or three days, a seton needle, half an inch broad, armed with a double ligature, was passed through the tongue from below upwards, cutting transversely; the ligatures were carried obliquely backward, and firmly tied on either side, so as to give to the remaining tongue a somewhat pointed appearance. The gush of blood on passing the needle through the tongue was very considerable, but almost instantly ceased on the ligatures being tied: twenty hours after the strangulated portion was removed by two strokes with a bistoury, from the centre outwards, in the course of the ligatures, which was followed by a few feeble jets of blood from the lingual arteries, and a slight oozing from the substance of the tongue for a few minutes. There was considerable irritation and constitutional disturbance after the application of the ligatures, which required a small bleeding and an anodyne; these symptoms all disappeared soon after the final excision.

The dressings consisted simply in the application of a pledget of lint moistened with a saturated solution of chloride of lime, renewed three or four times in the twenty-four hours, and a handkerchief tied over the mouth to protect it from the air. On the fourth or fifth day the child was walking about in the open air, and appeared to suffer very little: at the close of the second week the wound was nearly healed, and she was taken back into the country, with a request that she might be brought to Columbia again in four or five weeks; which was accordingly done. On her return, the wound was perfectly cicatrized, and the body of the tongue reduced to its natural dimensions. She had full command of her lips; and it was very evident that the jaws would soon be brought into their proper relation to each other by the natural action of the muscles. She could articulate with sufficient distinctness, and give all the letters of the alphabet their proper sounds.

Four months after this her father called on me, and said that his daughter had fully recovered, the jaws had lost their curvatures, all the teeth coming in contact. Mr. R. called on me again a few weeks since, and informed me that his daughter enjoyed good health, had felt no symptom of the old complaint in her tongue since her recovery from the operation.

---

### *Extirpation of the Testes and Penis, affected with a Cancerous Disease.* By J. C. Hall, M.D. (*Ibid.*)

The following case, given from memory, illustrates the propriety of pursuing with the knife a disease of the most malignant nature, and threatening a rapid destruction of life.

Mr. S. in mounting his horse many years ago, injured the left testicle, which, after causing much pain, dwindled away. Eighteen months since, this testicle grew irritable, painful, and much enlarged. It resisted every attempt at dispersion, and, occasioning much constitutional derangement, reduced him to the lowest extremity. In the fall of 1830, I saw him in consultation with Drs. Dawes and Causen. His aspect was bloated; limbs œdematous, and the lower extremities very painful from the distention; his strength was prostrated, and he might almost be pronounced moribund. The disease of the testis had burst through the inflamed and almost gangrenous scrotum, and was pouring forth a most luxuriant mass of fungus, easily bleeding, and emitting a most horrid stench. Much of this fungus had been clipped away by the patient and his surgeon, but still it was rapidly reproduced. As there was no alternative but immediate death, or an operation, the latter was determined on, but with no other expectation but that of deferring the fatal event. The patient having been revived by cordials, the testicle and all the affected portion of the scrotum was removed. The diseased mass, upon examination, appeared to arise rather from the tunics of the testicle than from the gland itself. Mr. S. was near sinking after the operation, but finally rallied, and the wound continued in a healthy condition for seven or eight weeks, when it assumed a malignant appearance, presenting all the indications of cancerous action. Defying every effort to arrest its progress, the knife was again resorted to, and the remaining testicle and much of the scrotum, was extirpated, embracing every diseased portion. Again the wound did well for some time, but, putting on the same threatening appearance, all the remaining scrotum was cut away, together with a portion of the perineal surface which had become affected.

These several operations were thought at the time to embrace every diseased part, and even more, but the wounds never healed, and finally, after the lapse of a year from the first operation, the disease recurred, and spread over the perineal integuments and the posterior inferior part of the penis, destroying the urethra to the extent of an inch, and affecting the lateral parts of the corpora cavernosa.

Though despairing of success, yet, at the urgent request of the patient, a final operation was decided on. Carrying the scalpel deeply through the integuments and fat upon the pubis, we divided the suspensory ligament and the fascia coming from the thigh to the penis. Thus detaching it from the pubic arch, we dissected its crura from the pubic rami, and divided them separately.

The urethra was divided at a very short distance from its exit through the triangular ligament. The operation was completed by carrying the knife beyond all the diseased parts visible, and excavating a large portion of the perineal surface. Eight vessels were tied, as necessarily cut, and no secondary hemorrhage of importance occurred. The wound left was very large, and the most soothing application was found to be fresh cream. The cure has been effected rapidly, perfectly, and we trust permanently.

Whether the disease with which Mr. S. was affected, was truly cancerous or not, does not depreciate the lesson of perseverance thus taught. His disease was rapidly hurrying him to his end, and had not his resolution given boldness to the surgeon, he would have been abandoned to a most horrid death.

---

### MEDICAL ELECTRICITY.

The following experiments appear so truly wonderful that, but for the good authority by which they are supported, I should feel unwilling to give them a place here. The remarkable results which accompanied them can scarcely be explained as coincidences, and they appear to open the way to new and important inquiries; but numerous observations and experiments must be made before we can be warranted in drawing any certain conclusions on the subject. P. Smith, of Fordham, to whose experiments I have already alluded, having relieved numerous patients, labouring under gout, rheumatism, and other painful affections, was induced to try the effects of electricity upon intermittent fevers. He intended to employ it as the cold stage was coming on, but his first patient having mistaken the time of the accession of the fit, he did not apply it until the hot stage had commenced: he insulated the man, and caused him to receive sparks at the epigastric region, while he took them from him along the course of the spine; the pulse was speedily reduced thirty beats per minute. The next patient was electrified on the coming on of the chill, which it immediately checked; nevertheless the hot stage ensued, when electricity was again applied, and, as in the former case, it reduced the pulse thirty beats: there was no return of the paroxysm, but the application of electricity was repeated for some days. Another case of ague, which had lasted four months, and obstinately resisted bark, arsenic, and other medicines, was quickly cured by a few applications to electricity. The most extraordinary circumstance remains to be mentioned. P. Smith himself had held the ball with which he took sparks from his first patient, during the hot stage. In the same evening, he found himself unwell; but had no suspicion of the nature of his complaint, until the recurrence of the paroxysm convinced him that he had become the subject of ague. He allowed these to recur to the seventh time, before he attempted the cure by electricity, which was speedily effected, being the second case already alluded to. As he had never been the subject of ague, and had not been more than usually exposed to causes calculated to give rise to it, he felt persuaded that it had been communicated to him by electricity from his former patient.

In order to ascertain this, he was desirous of trying experiments on some persons labouring under a disease which was inflammatory, but not considered infectious; he, therefore, had one of his men vaccinated. On the seventh day, the man was placed on the insulating stool, and connected with the positive conductor; a small incision was made with a lancet in the pustule, and an incision was also made in the arm of a lad with a new lancet; a wire four inches long was passed through a glass tube, one end of which touched the pustule on the man's arm, and the other the incision on the boy's arm, the electrification was continued for eight minutes, when the boy was removed. His arm was daily examined, and it was found that he was as completely vaccinated by electricity as any person could be by the usual mode. My friend afterwards endeavoured to communicate the virus to two girls, by passing the electrical fluid from the pustule on the boy's arm, who had been vaccinated by electricity, to incisions made in theirs. For three days the medical gentleman supposed it had taken effect; but, on the fourth day, all appearances of vaccination died away. These girls were, however, afterwards vaccinated in the usual way in four places, two of which died away, and the other two took but very slightly. My friend Charles Woodward afterwards repeated this experiment upon an infant, the child of one of his friends, but with this difference, that he did not allow the conducting wire to come in contact with the child's arm. The electric fluid was consequently transmitted in the form of small sparks. The disturbance which these produced, though trivial, prevented the application from being prolonged for the full time, which my friend would have wished: inflammation however succeeded, and, until the sixth day, was such as to induce the medical attendant to believe that the vaccination had been complete; from that day, however, the pustule died away.

Circumstances, which it is wholly foreign to this work to relate, interrupted the continuance of the research. The facts already related have come to my knowledge at too late a period to allow of my pursuing the investigation; but I trust it will not long continue neglected.—*From an Essay on some of the Phenomena of Atmospherical Electricity, by* Luke Howard, f.r.s., *in the Appendix to* Dr. Edwards *on the Influence of Physical Agents on Life.*

---

### MISCELLANEOUS.

*To the Editors of the London Medical and Physical Journal.*

Gentlemen: The very singular case of a patient in the Hôtel Dieu, recorded in the February number, p. 153, gives rise to a variety of curious speculations and inquiries relative to the cause of his paralysis.

1. "A slight eruption had followed the wound of the finger, in the form of pustulous pimples." It is not stated on which part of the body these appeared, or of what the matter was composed; but that they had disappeared when he was admitted into the hospital. Query. Might not this have been an effort of nature to relieve herself of some aqueous or serous humour, which, having been perhaps checked by cold or improper treatment, might be repelled, and thus produce a serous deposition or effusion in or on the spinal chord, from whence the nerves of motion take their rise?

2. "The paralysis commenced in the lower extremities and on the right side, and extended to the trunk, and, lastly, to the chest and upper extremities affecting the lungs partially, but not the diaphragm." Here we may observe, that it commenced at the greatest distance from the heart, as the source of circulation, and gradually extended towards it, affecting the lungs partially, and probably even the heart itself. From the limbs being free from pain, stiffness, or convulsive movements, it appears that the muscles were not affected, but the nerves both of motion and sensation. It is here a singular circumstance that the organs of sense, as seeing, hearing, smelling, and tasting, were not affected; and it, therefore, appears that the nerves of sensation from the brain, which supply these organs, were in a state of healthy action.

I had, during the last summer, the opportunity of seeing the following case in Islington workhouse, which I here give from notes taken at the time. The patient had lost all power of sensation, and apparently of speech, whether through deafness or dullness in the auditory nerves could not be determined. The most singular fact in this case was, that in whatever position his limbs were placed, so they remained, but without any rigidity, as they might be altered at the pleasure of those who placed them. He therefore appeared rather to have lost the powers of *volition* than those of *motion*. The nurse said he would often remain in the same position for one or two hours, and sometimes even longer.

Mr. Semple, in my presence, tried the following experiment: He raised one arm, the patient standing equally on both feet. On our return, after having gone round the wards, the nurse said he had stood thus three-quarters of an hour, and then fell back on his bed. Mr. S. then raised both arms: the patient stood on his feet, his legs were rather twisted round. He had held in his right hand, for some hours, a short pipe filled with tobacco, in the position of one who has just filled a pipe for smoking, with the forefinger placed on the bowl to keep in the charge. In this position he was placed, at a quarter past eleven A.M., and in less than a quarter of an hour after, just as we had left him, he gave a sudden scream, and fell back on his bed. His pulse was slow, but regular; his breathing almost imperceptible; and he seemed perfectly unconscious of any surrounding objects. He could walk about the yard slowly, without assistance, but was always watched. He generally sat on the side of his bed nearly all day, without speech or motion.

I know not what became of this case. His age, I think, was about thirty.

E. G. Ballard.

*Islington; 5th February*, 1833.

---

*Preservation and Reproduction of Leeches. Important Discovery*. M. Moreau, of Angers, has communicated to the medical journals the important discovery, made by M. Battu, lieutenant in the revenue police, at St. Seurin, of a new and effectual method of preserving these valuable animals. In consists in placing them in a box, about three feet square, half filled with layers of rich homogeneous French soil. At the bottom of this box is inserted a small plate of tin, pierced with minute holes, and the top is closed with linen in order to prevent the escape of the leeches. The earth is moistened with water every eight days. By this process he has preserved the same leeches several months, and has even seen them reproduce. In a second letter on this subject, M. Moreau states the results of some of his own experiments on the matter. Twelve leeches were placed in one of these boxes several months since, all in a state of emaciation and debility from protracted abstinence. On examining the box a few days since, nine of the leeches were found in full health, increased in size, and there was also found a great number of ova, and minute full-formed leeches produced iu the box itself. The earth proper to be employed is of a reddish-brown colour, and possesses a strong power of imbibition. It must not lie dry, pulverulent, or be mixed with the roots of grass, small stones, bits of wood, &c. The temperature of the place, too, M. Moreau deemed of importance to be taken into consideration. In the successful experiment now detailed, the temperature was maintained at about 50° Fahrenheit.

The druggists who trade with our East and West Indian possessions, would do well to submit these important facts to immediate experimental investigation.—*Lancet.*

---

CHEMISTRY.

*Composition of the Blood in Jaundice.* My friend, Dr. Corrigan, having transmitted to me a specimen of blood taken from a woman labouring under jaundice, I undertook the analysis, in order to verify the researches of Lecanu, and some analyses made by myself at a former period.

The blood had separated perfectly; the clot, not buffed, lay soft and loose at the under surface. The superficial arterialization was very imperfect, (the vessel had been closed air-tight by bladder.)

The serum was of a deep yellow colour, browned turmeric paper, was salty to the taste, and, when mixed with muriatic acid, became vivid grass green in colour. Sp. gr. 1029.25.

The quantity of blood sent to me weighed 3168 grains, consisting of

| | |
|---|---|
| Serum | 1420 |
| Clot | 1748 |

A quantity of serum having been carefully dried, was found to consist of

| | |
|---|---|
| Water | 899.1 |
| Solid matter | 106.9 |
| | 1000.0 |

A quantity of clot having been dried, was found to be composed of

| | |
|---|---|
| Water | 656 |
| Solid matter | 344 |
| | 1000 |

The salts were found, by appropriate re-agents, to be present in their natural proportion. Their quantitative estimation was not, in the future steps of the analysis, attempted.

The dried serum, having been reduced to very fine powder, was boiled in dilute alcohol, and afterwards in very strong alcohol. The alcoholic solutions were mixed together, and very much concentrated: they deposited successive crops of common salt, and were alkaline. They were finally evaporated to dryness.

The dried serum, when it ceased to yield any soluble matter to boiling alcohol, was weighed and estimated as albumen.

The dry residue of the alcoholic solutions was digested in cold ether, which dissolved most of it, and assumed a gold colour. The undissolved matter was boiled in a small quantity of alcohol, which dissolved it, and, on cooling, deposited flocculi of phosphuretted fat. The ethereal solution, when evaporated, gave a residue of the yellow biliary colouring matter, and an oily liquid which floated quite separate from it; (*oil of Babington.*)

The clot was washed with water carefully until the fibrine remained white; it was then carefully dried and weighed.

The liquor in which the clot had been washed was heated to 200°; the mixture of albumen and hematosine coagulated, and the quantity of the former having been gotten by calculation from the quantity of water that existed in the clot, as serum, and added to the albumen before obtained, we have the entire quantity of albumen in the blood, and the residue gives, of course, the colouring matter.

In former analyses of the blood in disease, I usually icinerated the hematosine, in order to determine the quantity of oxide of iron that I could obtain: but, as the iron exists in the hematosine, not as oxide, but probably as an ultimate constituent, and is consequently proportional in quantity to that of the hematosine, I do not now think it necessary to determine its amount separately.

The quantitative results of the analysis are as follows:

| | |
|---|---|
| Water | 762.28 |
| Albumen | 71.4 |
| Fibrine | 9.8 |
| Hematosine | 126.7 |
| Phosphuretted Fat / Oily matter and yellow colouring matter | 2.0 |
| Salt, loss, &c. | 34.82 |
| | 1000.00 |

This analysis fully confirms the result of Lecanu as to the existence, in the blood of jaundiced patients, of the yellow colouring matter of the bile, and also as to the absence of cholesterine. In consequence of this latter principle being often a constituent, in minute proportion, of healthy blood, I sought for it carefully, but could not detect any trace of it. My results, however, differ in an important relation from those of Lecanu: he found that the quantity of colouring matter was less than in health; in the foregoing analysis, it is very nearly the exact healthy average. I am not inclined to attribute great importance to the difference between our results in this respect, as the quantity of that principle varies in health between limits extending beyond both.—Mr. Kane; *Dublin Journ. of Med. and Chem. Science.*

### BOTANY.

*Secretion of Water by certain Plants.* Professor I. C. TREVIRANUS, of Breslau, has lately given his attention to the subject of the watery secretion of the leaves of plants. The Nepenthes, Sarracenia, and Cepholatus have long afforded the most striking examples of this function. Rumple's observation, that the water of the tankard-shaped leaves of the N. distillatoria is always pure, militates against the supposition that it comes from without. He also remarked that when the lid of the N. Phyllamphora is open, the water is diminished one half by solar evaporation, which, however, is restored at night. The structure of the goblet-like leaf, as observed by Treviranus, is like an actual secreting organ, and adds a strong reason for thinking that the plant supplies the water. He finds the parietes of the leaf of N. distillatoria traversed by a multitude of proportionably-large anastomosing veins, which contain many true spiral vessels. The upper half of its inner surface is covered with a blue rind, as parts often are which are to be protected from the action of water; the under half is, on the contrary, shining and full of small gland-like eminences, directed downwards, and having a hole almost visible with the naked eye, which is uncovered with the cuticle which the remainder possesses. Through these, he thinks, is the water secreted, and it reaches generally to their level in the middle of the leaf: it is remarkable, that the inner or under surface of the lid exhibits a similar structure, but whether it also secretes water future observation must discover. Sir J. Smith's remarks on the construction of the lid of the Sarrencia flava and adunca are sufficient to invalidate Linnæus' opinion, that the leaves of this genus, as well as the nympheæ, were intended as natural reservoirs for rain-water. In the Sarracenia there is no particular apparatus, as in the Nepenthes, for the secretion. Macbride's observations demonstrated, on the edge of the Sarracenia adunca, a sweet substance that allures insects, which, creeping into the funnel of the leaf, arrive at the water, and, being hindered from returning by the hairs directed downwards, are drowned. It is reserved for future investigation, to discover what gives the sweet taste to the Nepenthes and Cephalotus which is mentioned, and how the insects are killed, as nothing appears to hinder their creeping out again.

Treviranus has examined particularly the watery secretion of the Amomum Zerumbet, which was not noticed by any botanist, except cursorily by Murray. The spikes stand on a stalk a foot and a half long, rising from the root, and are the size of a hen-egg, or sometimes as large as a goose-egg; they consist of a great number of broad deep scabs which lie over one another, imbricated, and enclosing a space between them. The scales are of a leathery consistence, each of them enclosing a small colourless flower, of a more cuticular nature. At the commencement of the flowering, the spikes are full of clear water, which is nearly without smell or taste. By gentle pressure, it comes from between the scales, and, if it be emptied in the evening, it becomes in great part renewed by morning. The lower half of the scale, which contains no flower, is found as full of water as the rest. Treviranus, therefore, considers the inner inferior part, where the scales are connected with the stalk, the place where the water proceeds from. The water lasted during the whole flowering time, that is three weeks, but as it advanced, it did not preserve its original pureness; it became somewhat ropy, and got the smell of the bruised leaves of the plant, without, however, losing its transparency in the least. Dr. Goppert subjected a portion of it to chemical analysis, from which it results that the fluid between the scales of the spikes of the Amomum Zerumbet consists of pure water, containing a small quantity of vegetable fibrine and mucus; the quantity of which last is different at different periods of the flowering time.

He has also observed a tasteless water in the corolla of Moranta gibba. —*Treviranus und Tiedemann, Zeitschrift;* and *Dublin Journal of Med. and Chemical Science.*

### INTELLIGENCE.

